# New Acquisition Protocol of ^**18**^F-Choline PET/CT in Prostate Cancer Patients: Review of the Literature about Methodology and Proposal of Standardization

**DOI:** 10.1155/2014/215650

**Published:** 2014-07-10

**Authors:** Sotirios Chondrogiannis, Maria Cristina Marzola, Gaia Grassetto, Anna Margherita Maffione, Lucia Rampin, Emma Veronese, Arianna Massaro, Domenico Rubello

**Affiliations:** Department of Nuclear Medicine, PET/CT Centre, “Santa Maria della Misericordia” Hospital, Via Tre Martiri 140, 45100 Rovigo, Italy

## Abstract

*Purpose*. (1) To evaluate a new acquisition protocol of ^18^F-choline (FCH) PET/CT for prostate cancer patients (PC), (2) to review acquisition ^18^F-choline PET/CT methodology, and (3) to propose a standardized acquisition protocol on FCH PET/CT in PC patients. *Materials*. 100 consecutive PC patients (mean age 70.5 years, mean PSA 21.35 ng/mL) were prospectively evaluated. New protocol consisted of an early scan of the pelvis immediately after the injection of the tracer (1 bed position of 4 min) followed by a whole body scan at one 1 hour. Early and 1 hour images were compared for interfering activity and pathologic findings. *Results*. The overall detection rate of FCH PET/CT was 64%. The early static images of the pelvis showed absence of radioactive urine in ureters, bladder, or urethra which allowed a clean evaluation of the prostatic fossae. Uptake in the prostatic region was better visualized in the early phase in 26% (7/30) of cases. Other pelvic pathologic findings (bone and lymph nodes) were visualized in both early and late images. *Conclusion*. Early ^18^F-choline images improve visualization of abnormal uptake in prostate fossae. All pathologic pelvic deposits (prostate, lymph nodes, and bone) were visualized in both early and late images.

## 1. Introduction

Prostate cancer (PC) is the most frequent cancer among men and the third cause of death in economically developed countries [1]. As for every neoplasm precise initial staging and early identification of recurrence are important factors for an adequate treatment of primary and recurrent PC.

In recent years functional PET/CT imaging using radiolabeled choline has been extensively studied for imaging PC, gaining an important role on its management, especially in restaging patients with biochemical relapse only after radical treatments.

Choline can be labeled with both 11C and ^18^F. The use of 11C-choline is limited in centres with an on-site cyclotron and radiopharmacy facilities, because of its very short half-life (20 minutes). The short half-life of 11C requires a short interval between synthesis, injection, and acquisition and is less suitable for late imaging. On the other hand, 11C-choline shows low renal excretion, resulting in accurate detection of recurrences in the prostatic fossae and loco regional lymph nodes [2–4].

^18^F-Choline (FCH) is becoming more widely available (it can be prepared on a commercial basis and distributed to PET centres without a cyclotron unit on site) with a stable biodistribution and a longer half-life (^18^F half-life = 110 minutes), moreover allowing delayed acquisition also [5–7]. However, FCH presents a variable urinary excretion with high accumulation in the bladder that can compromise the evaluation of the prostatic region. Fortunately, when using FCH, delayed scans can be acquired, providing better quality imaging since the rapid washout of FCH leads to more favourable (higher) tumor-to-background ratios [5, 8].

Despite the promising results reported in the literature, there are still no definitive guidelines on choline PET/CT utilization, both regarding technical and clinical aspects. A variety of open questions remain to be solved as (a) at which trigger PSA value a choline PET/CT should be performed; (b) should antiandrogen therapy be withdrawn before performing choline PET; (c) is biochemical relapse the only clinical indication for choline PET/CT; (d) what preparation should be suggested to the patient; (e) which acquisition protocol should be adopted: that is, timing of the scanning (early, delayed, or both) and acquisition modality (dynamic, segmental, or whole body). Recent reviews and meta-analyses of the literature have been focused on the results of choline PET/CT reporting very interesting rates of sensitivity, specificity, accuracy, positive, and negative predictive values (PPV, NPV); however, no guidelines are still available for the utilization of choline PET, and acquisition protocols often differ from a centre to another [9, 10] (see Table 1).

The purposes of our study were (1) to evaluate the usefulness and feasibility of a static acquisition of the pelvic region performed immediately after tracer injection of FCH PET/CT in order to obtain a clean visualization of the prostatic fossae, thus avoiding interference of radioactive urine, followed by a standard whole body scan one hour p.i.; (2) to review the literature on acquisition methods used with FCH PET/CT for prostate cancer patients; and (3) to propose a standardized acquisition protocol on FCH PET/CT.

## 2. Materials and Methods

### 2.1. Patients

We have prospectively evaluated FCH PET/CT scans of 100 consecutive patients (mean age 71 years, range 41–87) with prostate cancer who were referred to our centre to perform FCH PET/CT from November 2012 to May 2013. Of them, 70 were treated with radical surgery and/or radiotherapy, and 18 were under antiandrogen treatment (ADT) only; lastly, 12 patients had not received any type of treatment. Mean trigger PSA was 21.35 ng/mL (range 0.1–319 ng/mL) measured within one month before the PET scan.

All patients were asked for written consent to use the results of their examinations for the purposes of the present study.

### 2.2. Acquisition Protocol

Patients fasted for 6 hours before the PET scan. At arrival to our department they were asked to drink 500 mL of water and to avoid emptying the bladder before the first part of acquisition. All FCH PET/CT scans were obtained with a hybrid PET/CT tomograph (General Electric, Discovery STE, Milwaukee, USA).

The PET/CT acquisition protocol consisted of a dual phase procedure: a static acquisition of the pelvis obtained immediately after tracer injection (1 bed position of 4 min) followed by a whole body scan 60 minutes after injection from the base of the skull to the superior portion of the thighs (6-7 bed positions, 3 minutes per bed) with patient positioned supine with arms crossed above head. The early phase was performed in order to avoid possible interference of radioactive urine in the excretory pathways: to this purpose the patients were positioned on the PET bed, localizing the laser alignment system on the iliac spine, without voiding the bladder (bladder full of “cold” urine) and PET acquisition started immediately after tracer injection (3 MBq/kg/body weight of ^18^F-Choline; IASOCholine, ARGOS Zyklotron GmbH, Linz, Austria), in order to have the minimum time gap between PET acquisition and tracer injection. Whole body scan was acquired 1 hour after injection and patients were invited to empty the bladder.

Each CT acquisition was preceded by a scout-view (80 kV, 10 mA, anteroposterior) to set PET/CT scan limits: in the static acquisition of the pelvis, the scan superior border coincided with the upper portion of the iliac bone and the pubic symphysis had to stay on the scan centre, in order to include prostate and regional lymph node, including the inguinal ones. For the whole-body acquisition, the scan ranged from the orbitomeatal level to the superior portion of the thigh. Two types of CT scans were acquired: the helical CT scan of the pelvis (120 kV, 80 mA, slice thickness 3.75 mm, pitch 1.375, speed 13.75 mm/rot, rotation time 0.8 s, and 50 cm FOV diameter) and the whole body helical CT scan (120 kV, 40–100 mA modulated by GE Smart-mA with a noise index of 30, slice thickness 3.75 mm, pitch 1.375, speed 13.75 mm/rotation, rotation time 0.8 s, and 50 cm FOV diameter). All PET scans were acquired in 3D mode, with FOV diameter 50 cm; data were collected in list mode and reconstructed by the VUE-Point (General Electric, Milwaukee, USA) fully-3D iterative reconstruction algorithm, with 20 subsets by 2 iterations, 128 × 128 matrix.

### 2.3. Image Interpretation

All FCH PET/CT scans were evaluated both by visual (using transaxial, coronal, and sagittal views) and semiquantitative analysis using the maximum standardized uptake value (SUVmax), on a patient-by-patient and lesion-by-lesion basis, by two nuclear medicine physicians (S.C. and M.C.M., both with an experience of more than 4 years in reading FCH scans) using a dedicated software (XELERIS workstation version 2.1753; General Electric, Milwaukee, USA) that allows review of PET, CT, and fused PET/CT images. In case of discrepancies, final diagnosis was reached by consensus. Any focus of nonphysiological uptake was considered as pathologic. CT images were used for both attenuation correction and topographic localization.

In order to assess the usefulness of the early static acquisition of the pelvis a comparison between early and 1 hour images of the pelvic region was performed by visual and semiquantitative analysis evaluating:

(A) activity in the urinary tract (bladder, ureters, and urethra) and whether it could interfere with the reading of the scan, especially focused to the prostatic bed, using a 3-scale score (0: no accumulation, 1: mild accumulation not interfering with the reading, 3: intense accumulation that could interfere with the reading);

(B) pathologic findings in lymph nodes and bone included in the field of view (pelvic region 1 bed position).

The purpose was to assess whether the new static acquisition of the pelvis obtained immediately after tracer injection would be able not only to avoid the radioactive urine interference in the prostatic bed but also to identify pathologic localizations of the disease using the late images as reference.

Positive PET/CT findings were validated on a patient basis by TRUS-guided biopsy or surgical lymphadenectomy in 42 patients and by other imaging procedures including CT and MRI or clinical, laboratory (PSA) and imaging follow-up including control ^18^F-CH PET/CT in the remaining 58 patients.

### 2.4. Review of the Literature

We also performed a computed literature research about studies in clinical setting of prostate cancer using as key words: ^18^F-choline or fluorocholine PET or PET/CT and prostate cancer. The PubMed database was used considering papers on ^18^F-choline utilization on prostate cancer independently from the purpose of the study (staging, restaging, etc.). Literature review was based on methodology and acquisition protocol of  ^18^F-choline PET and included papers in English language, published between 2006 and 2014 with well defined and described acquisition procedures (Table 1).

## 3. Results

In our series the overall detection rate of FCH was 64% (64/100 patients): (a) 18/64 (28%) PET-positive patients showed only local pathologic uptake: in 13 cases confined to the prostatic bed, in 2 cases to the loco regional lymph nodes, and in 3 cases to both sites; (b) 20/64 (31%) showed distant localizations only (in 9 cases confined to the bone, in 13 cases to distant lymph nodes, and in 4 cases to both lymph nodes and bone). The remaining 26 patients (41%) showed both local and distant localizations.

**Figure 1:**
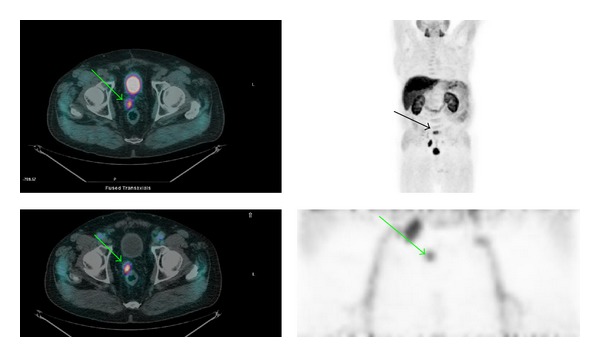
Figure shows an example of relapse in the prostatic region (green arrow) and how it is seen, respectively, in the delayed (upper row) and early images (lower row). Note in the early images the visualization of the iliac vessels, the absent accumulation of radioactive urine in ureters, bladder, and urethra, and the pathologic accumulation of the tracer in the prostatic fossa and the right iliac nodes. In the late images (Figure 1, MIP) a bone lesion has been also depicted in S1 outside the FOV (field of view) of the early images (black arrow). Upper left: delayed fused whole body PET/CT images. Upper right: delayed whole body MIP (multiple intensity projection) images. Low left: early fused PET/CT images. Low right: MIP images of the pelvic region (1 bed position).

In nearly all patients (99/100 of the cases) the early static acquisition of the pelvis showed absence of radioactive urine in ureters, bladder, or urethra which allowed a very clean evaluation of the prostatic region. In the late images, in 76% of the cases activity in the bladder could potentially interfere with the reading. Moreover, pathologic uptake in the prostatic region (present in 30 patients) was better visualized in the early images in 23% (7/30) of the patients. Other pelvic pathologic findings (bone and lymph nodes) were visualized both in the early and late images (Figure 1).

Review of the literature included 24 papers from 9 different countries on FCH PET or PET/CT for prostate cancer evaluation (2256 FCH PET or PET/CT scans) published between 2006 and 2014 (Table 1). There was a large variability on every aspect of the methodology adopted by each centre about the performance of FCH PET. Preparation before presenting for FCH PET ranged from a fasting condition of 12 hours [12], avoidance of foods containing high levels of choline for the week before the scan [26] to “fasting no necessary” [19] with also variable instructions on liquid assumption before scanning ranging from avoidance of liquids 1-hour before presenting to the PET department to reduce bladder filling before tracer injection [7], to hydration with 1.5 lt of water enriched with oral contrast 1-hour before injection [14]. Injected dose was also very variable ranging from fixed dose to dose calculated per body weight (ranging between 2.6 and 4.07 MBq/kg). The higher variability though regards acquisition protocols. In 10/24 papers only one phase whole body acquisition is described at 2 min [13, 15], 12–15 min [21], 45 min [26], 60 min [6, 20, 23, 27, 30], and 60–90 min [24] with variable duration per minute of bed position (ranging from 2 to 7 min). The rest of the papers (14/25) describe at least two point acquisition protocols [5, 7, 8, 11, 12, 14, 16–19, 22, 25, 28, 29]. Of them; 7 have adopted dynamic acquisitions of the pelvic region just after the injection of the tracer [8, 12, 16, 17, 19, 25, 28] and 5 performed static acquisition of the pelvis of one or two bed positions with time range between 1–15 min p.i. [5, 7, 14, 22, 29], while other 2 papers describe whole or “partial” body early acquisitions [11, 18]. Three papers describe three-point acquisition protocols, with late scans after the whole body acquisition performed routinely [8, 28] or if needed [25, 26] (see Table 1).

## 4. Discussion

The concept of functional imaging is based on the molecular characteristics of the different pathologies. In recent years the introduction of new radiopharmaceuticals and new hybrid functional/morphological systems has expanded the indications of functional/molecular imaging in nearly every field of oncology. Functional imaging depends on (1) the radiopharmaceuticals, which need to be disease-specific, with favourable chemical and physical characteristics, (2) the technological evolution of the hybrid PET/CT system, which should provide the state-of-the-art on both hardware and software, and (3) the experience of the reader. A very important aspect of functional/molecular imaging involves the methodology of the acquisition that requires an adequate knowledge of the tracers characteristics, the pathology, the anatomy, the imaging methodologies, and the possible limitations.

In case of biochemical relapse in prostate cancer the PSA elevation indicates the presence of disease recurrence which is usually located to the prostatic bed, to adjacent or distant lymph nodes, and to bone. Patients are referred to PET/CT centres for choline PET in order to localize the site of relapse, to assess whether it is local or systemic, in order to choose the better therapeutic approach. The greatest advantage of choline PET/CT is that it is a whole body, noninvasive imaging modality capable of assessing the disease recurrence in multiple anatomical sites with higher accuracy than conventional imaging and FDG PET [2]. Even though FCH PET/CT is being increasingly used in clinical practice there are some limitations that need to be highlighted: (I) FCH is very promising for prostate cancer evaluation but not specific for prostate cancer only, (II) the hybrid machine (last generation PET/CT) has a spatial resolution of ≈0.6 cm which make it unsuitable for the detection of millimetric disease, and (III) because of the characteristics of the tracer and the anatomy of the pelvis we may have radioactive urine interference in the prostatic fossae. This paper is focused on the possible strategies that could be used to reduce the third limitation.

When using FCH PET/CT, the variable presence of radioactive urine in the urinary pathways and the interference that causes are known. Pelosi et al. in a study on 56 patients using a protocol that consisted only on delayed 1 hour whole body images report five false negative cases in the prostatic bed probably due to the urinary excretion of the tracer that can mask the small areas of pathological uptake at the prostate bed level [6]. In a recent meta-analysis on both 11C-choline and FCH, the main causes of the relatively low tumor recurrences detected with PET in the postsurgical prostatic bed (sensitivity 43%–83% and specificity 50%–91%) compared to other imaging techniques that (i.e., TRUS and MRI) were related to the limited size of recurrent lesions, the partial volume effect, and the presence of urine in case of FCH [9]. For this reasons an early phase acquisition step (dynamic or static) has been recommended in order to better evaluate (a) the prostatic fossae before the arrival of the radioactive urine in the bladder and (b) the pelvic lymph nodes [12]. This is possible because, as firstly described by DeGrado et al., the uptake of the tracer in malignant lesions occurs promptly, whereas radioactive urine in the urinary bladder appears later (approximately 5–8 min. p.i.) [31]. These biokinetic characteristics offer a time window in which the pathologic areas are visible, avoiding radioactive urine interference because urine has not yet arrived in the ureters, bladder, and urethra. Some authors prefer the dynamic acquisition to a static one because the earliest moment at which the tracer appears in the urinary bladder can vary considerably, and with the dynamic acquisition they can identify it [12].

Hara et al. solved the problem of renal elimination of the FCH by continuous bladder irrigation using a urinary catheter to eliminate the bladder radioactivity, procedure that is however very uncomfortable for the patients [32].

Additionally, some authors report that dual-phase PET improves differentiation between benign and malignant: areas of malignancy demonstrated stable or increasing FCH uptake over time, whereas benign areas showed a decreasing pattern [11, 17].

Similarly Hodolic et al. used an early phase acquisition to evaluate prostate or prostate bed and reported that early pathologic findings were also seen in the late phase. In patients with bone metastases in the pelvis the pathological uptake was depicted in metastases already during the first 5 min after the tracer injection. Time-trends of enhanced FCH uptake can help to characterize also pathologic lymph nodes in prostate cancer patients [22, 25]. Late imaging (at 65–200 min p.i.) may enhance the detection rate of bone metastases on FCH PET/CT scans [5]; moreover FCH accumulation in bone metastases on late imaging rises compared to early imaging (5–15 min p.i.) [15].

Some authors proposed a three-phase protocol for increasing the diagnostic assessment of local recurrence disease [8, 28], or delayed scans in case of equivocal findings (after deambulation, hydration, diuretic administration, etc.) [25].

Graute et al. using single phase protocol report superior sensitivity plausibly attributed to the use of high dose CT in conjunction with contrast agent. In 10 of 82 patients they studied the use of CT contrast agent that enabled the differentiation of physiological urinary activity and FCH uptake. Moreover, the use of contrast agent was also helpful for excluding the possibility of infiltration of adjacent organs such as the bladder and rectum. Furthermore, authors were able to identify small lymph node metastases with only marginal to moderate tracer uptake lying in close proximity to pelvic vessels or intestinal structures and they suggest to use contrast agent whenever possible in daily practice, because of the substantial benefits it brings to ease and reliability of PET interpretation [23].

Our group has already published a paper describing our experience to optimise the acquisition protocol for FCH PET/CT in prostate cancer patients that would be ideal not only from a clinical point of view but also for logistic reasons [33]. It consisted of several acquisition steps: (i) an early dynamic scan of the pelvis, (ii) an early static scan of the pelvis, (iii) an early whole-body scan, (iv) delayed static scan of the pelvis, and (v) delayed whole-body scan. The most useful information was provided by the whole body scan because both local recurrences and distant metastases could be depicted; its main limitation was the variable urinary excretion that could interfere with the reading. In this regard the static scan of the pelvis was very useful in some cases, allowing to distinguish foci of radioactive urine in the bladder and urethra from locoregional recurrences.

**Figure 2:**
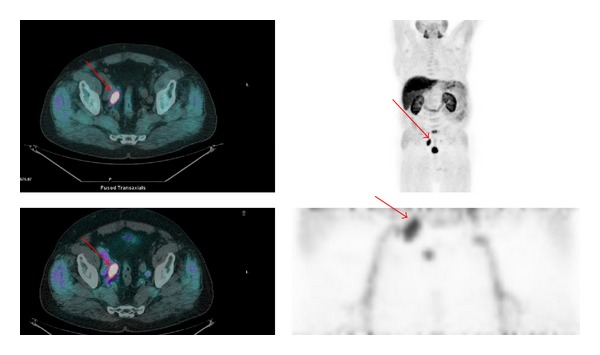
Figure shows, in the same patient as Figure 1, an example of relapse to a right iliac lymph node (red arrow) and how it is seen, respectively, in the delayed (upper row) and early images (lower row). Upper left: delayed fused whole body PET/CT images. Upper right: delayed whole body MIP (multiple intensity projection) images. Low left: early fused PET/CT images of the pelvis. Low right: MIP images of the pelvic region (1 bed position).

However the variable urinary excretion of FCH PET/CT often leads to interpretative doubts especially in the prostatic region. In order to avoid even the minimum presence of radioactive urine in the bladder we tested a new, as early as possible, static acquisition of the pelvic region obtained immediately after the injection of the tracer. The tracer is injected after the low dose CT, with the patient already positioned on the PET bed, in order to have the minimum time gap between injection and acquisition; moreover patients are positioned on the PET bed with bladder full of cold urine. Using this new protocol we have noticed in all patients absence of radioactive urine in the ureters, bladder, and urethra which allowed a clean reading of the whole pelvic region and helps to consider as suspicious any foci of focal uptake within the prostatic fossae or the pelvic lymph nodes (Figures 1 and 2).

Moreover as already described by other centres, PET findings in the early images are confirmed in the late images and the variation in terms of SUV can guide towards a malignant (if increases) or benign (if decreases) characterization of the abnormal uptakes [11, 17, 22, 25].

In conclusion, on the basis of the data of the present study, a “two-step” FCH PET/CT protocol based on early static pelvic acquisition immediately after tracer injection and delayed (at 1 hour after injection) whole body acquisition seems adequate enough to obtain good quality imaging, to visualize recurrent disease and at the same time to avoid false-positive or false-negative results related to urinary excretion. The early static pelvic acquisition allows to study the prostate region before physiological urinary excretion, while the delayed whole-body scan was useful to confirm the findings in the pelvis and to evaluate the presence of distant metastases.

## 5. Conclusions

Even though FCH PET/CT is being increasingly used in clinical practice for the evaluation of prostate cancer especially in patients with biochemical relapse, there are still no definitive guidelines about its utilization. Regarding methodology, a known limitation of FCH PET/CT is mainly the variable urinary excretion of the tracer that can interfere with the reading of the pelvic region, especially the prostatic fossae. This limitation can be overcome by an early acquisition (static or dynamic) of the pelvis before the arrival of the tracer in the urinary pathways. For this reason standard acquisition protocol of FCH PET/CT should include an early acquisition of the pelvis (dynamic or static, as early as possible after the injection of the tracer) followed by a whole body scan. Whole body acquisition evaluates both local and distant pathologic deposits of FCH, while early acquisition of the pelvis offers a clean visualization of the pelvis itself, especially of the prostatic fossae, and helps reach final diagnosis of local relapse with more confidence, without any radioactive urine interference.

## Figures and Tables

**Table 1:** The results of the acquisition protocols for ^18^F-Choline PET/CT in prostate cancer patients used in the published papers in the literature described in detail.

	Author	Country	Year of publication	Number of patients	Indication for ^18^F-choline PET	Dose of ^18^F-choline	Early static acquisition: timing, FOVs time per FOV	Early dynamic acquisition	Delayed acquisition	Other delayed acquisitions	Transmission scans	Patients preparation
1	Kwee et al. [11]	USA	2006	26		3.3–4 MBq/kg	7 min p.i. 5 FOVs, 7 min per FOV		60 min p.i. pelvic region 1 bed of 7 min		^ 68^Ge rod sources	

2	Heinisch et al. [12]	Austria	2006	45	Restaging	4.07 MBq/kg		8 min dynamic PET in the pelvic region (1 min frames) 1 min p.i.	14 to 19 min p.i. (mean 17,6 min p.i.)		Low dose CT	Fasting for 12 hours

3	Cimitan et al. [5]	Italy	2006	100	Restaging	3.7–4.07 MBq/kg	5–15 min p.i. (2 FOVs, 5 min/FOV)		65–200 min p.i. (6-7 bed positions, 5 min per bed)		Low dose CT	Fasting for 6 hours

4	Vees et al. [13]	Switzerland	2007	11	Restaging	214 ± 14 MBq (no adjustment for weight or size)	2 min p.i. (7 FOVs, 3 min/FOV)				Unenhanced low dose CT scan	

5	Igerc et al. [14]	Austria	2008	20	Psa elevation negative biopsy	4 MBq/kg	3–5 min p.i. (1 FOV, 2 min/FOV)		30 min p.i. whole body 2 min per bed		Contrast enhanced CT	1.5 lt of water + oral mdc 1 hour before injection

6	Husarik et al. [15]	Switzerland	2008	111	Staging + restaging	200 MBq	2 min p.i. (6-7 FOVs, 3 min/FOV)					

7	Pelosi et al. [6]	Italy	2008	56	Restaging	185–259 MBq			60 min p.i. (mean 7 FOVs, 3.5 min per FOV)		Low dose CT and oral administration of 10 mL of contrast medium in half a litre of water	

8	Steiner et al. [8]	Germany	2009	47	Restaging	300 MBq		10 minutes list-mode PET acquisition over the prostate bed p.i. Three timeframes of 3 minutes each were reconstructed for analysis.	10 min p.i. whole body PET/CT	Delayed pelvic PET/CT		

9	Beauregard et al. [16]	Australia	2010	16	Staging − restaging	median 188 MBq (114–215 MBq)		10 min dynamic acquisition of the pelvis at time of injection, 1 min per frame, 1 bed position	15 min, 5-6 bed positions, 5 min per bed.		Low dose CT	

10	Hodolic [17]	Slovenia	2011	50	Staging + restaging	200–300 MBq		5 min list mode acquisition over prostatic bed immediately after the injection	60 min p.i. (9 bed positions on average) 2 min per bed			Fast 6–10 hours prior the scan

11	Soyka et al. [18]	Switzerland	2012	156	Restaging	200–300 MBq	partial wb 3-4 min p.i.		15–20 min p.i. partial body scan without taking the patient off the examination table and without voiding of the bladder		Low dose CT no contrast medium	

12	Henninger et al. [19]	Austria	2012	35	Restaging	4 MBq/kg		8 min (1 min per frame) dynamic emission scan of the pelvis 1 min p.i.	Whole body scan after the dynamic scan (7–9 FOVs, 5 min/FOV)		Germanium-67 rod source,	Fasting was not essential

13	Poulsen et al. [20]	Denmark	2012	210	Staging	4 MBq/kg			60 min p.i. wb scan (base of the skull to mid-thigh), 2.5 min/bed		Diagnostic CT with contrast	Fasting for 6 hours

14	Kwee et al. [21]	USA	2012	50	Staging − restaging	2.6 MBq/kg			12–15 min p.i., wb 9–11 FOVs, 2 min/FOV			Fasting for at least 3 hours

15	Oprea-Lager et al. [22]	Netherlands	2012	25	Staging − restaging	4 MBq/kg	pelvic region 2 min p.i (2 min/FOV)		30 min p.i. mid-thigh to the skull vertex, 2 min/FOV		Low dose CT	Similar to that required for FDG PET. Empty bladder before late scan

16	Graute et al. [23]	Germany	2012	82	Restaging	mean dose 300 MBq, normalized to body mass.			60 min p.i. 3 min/FOV		Diagnostic CT unenhanced	Prior wb scan pts were asked to empty their bladder so as to minimize tracer accumulation.

17	Kjölhede et al. [24]	Sweden	2012	174	Staging	4 MBq/kg (max dose 400 MBq)			60–90 min p.i. wb PET (pelvis to neck), 2 min/FOV		Diagnostic quality CT with contrast medium	Fasting for 4 hours before tracer injection

18	Marzola et al. [7]	Italy	2013	233	Restaging	3 MBq/kg	5–10 min p.i. static acquisition of the pelvis (1 FOV of 4 min)		60 min p.i. wb scan from the orbitomeatal level to the superior portion of the thighs (6-7 FOVs, 3 min/FOV)		Low dose CT no contrast	6-hour fasting and 1-hour avoidance of liquids to reduce bladder filling before tracer injection. Voiding was requested immediately before scanning to minimize the presence of tracer in the urinary tract.

19	Beheshti et al. [25]	Austria	2013	250	Restaging	4.07 MBq/kg		8 min dynamic PET images of the pelvis (1 min/frame) 1 min p.i.	10 min (6-7 FOVs), 4 min/FOV	If abnormal tracer uptake further delayed static image at 90–120 min	Low dose CT + diagnostic CT in 60% of the pts	

20	Calabria et al. [26]	Italy	2014	300	Staging − restaging	300 MBq range, 240–340 MBq			45 min p.i. wb scan, 5-7 FOVs, 3 min/FOV	Delayed if needed	Low dose CT + diagnostic CT, unenhanced	Fasting for at least 6 hours, on the week before diet avoiding foods containing high levels of choline.

21	Afshar-Oromieh et al. [27]	Germany	2014	38	Restaging	3 MBq/kg.			60 min p.i 4 min/FOV		Low dose CT no constrast medium	

22	Buchegger et al. [28]	Switzerland	2014	23	Restaging	307 ± 16 MBq		Tracer injection after the CT scan. 10 min list-mode acquisition on the prostate bed starting immediately with the tracer injection generating 3 × 3-min time frames corresponding to 0 to 3 min, 3 to 6 min, and 6 to 9 min after injection.	10 min after injection standard wb 7-8 FOVs, 3-4 min/FOV each depending on patient size and weight.	Two additional late images of 5 min each of the pelvis immediately after the wb PET scan (about 45 min after tracer injection).	Low dose CT	Fasting for at least 4 hours before the FCH. Empty bladder on scanning.

23	Detti et al. [29]	Italy	2013	129	Restaging	3.7 MBq/kg	Pelvic region 1 min p.i. 2-3 FOVs, 2 min/FOV		60 min p.i. 8-9 FOVs, 2 min/FOV			Fasting for 6 hours before tracer injection

24	Hausmann et al. [30]	Germany	2014	32	Restaging	mean 345 ± 24 MBq			60 min p.i. 8 bed positions			
